# Is cycle network expansion cost-effective? A health economic evaluation of cycling in Oslo

**DOI:** 10.1186/s12889-020-09764-5

**Published:** 2020-12-07

**Authors:** Admassu N. Lamu, Abdulrahman Jbaily, Stéphane Verguet, Bjarne Robberstad, Ole Frithjof Norheim

**Affiliations:** 1 Department of Global Public Health and Primary Care, University of Bergen, Post box 7804, N-5020 Bergen, Norway; 2 Department of Global Health and Population, Harvard T.H. Chan School of Public Health, Boston, MA USA

**Keywords:** Cycling network, QALY, Economic evaluation, Markov model, Cycling, Physical activity

## Abstract

**Background:**

Expansion of designated cycling networks increases cycling for transport that, in turn, increases physical activity, contributing to improvement in public health. This paper aims to determine whether cycle-network construction in a large city is cost-effective when compared to the status-quo. We developed a cycle-network investment model (CIM) for Oslo and explored its impact on overall health and wellbeing resulting from the increased physical activity.

**Methods:**

First, we applied a regression technique on cycling data from 123 major European cities to model the effect of additional cycle-networks on the share of cyclists. Second, we used a Markov model to capture health benefits from increased cycling for people starting to ride cycle at the age of 30 over the next 25 years. All health gains were measured in quality-adjusted life years (QALYs). Costs were estimated in US dollars. Other data to populate the model were derived from a comprehensive literature search of epidemiological and economic evaluation studies. Uncertainty was assessed using deterministic and probabilistic sensitivity analyses.

**Results:**

Our regression analysis reveals that a 100 km new cycle network construction in Oslo city would increase cycling share by 3%. Under the base-case assumptions, where the benefits of the cycle-network investment relating to increased physical activity are sustained over 25 years, the predicted average increases in costs and QALYs per person are $416 and 0.019, respectively. Thus, the incremental costs are $22,350 per QALY gained. This is considered highly cost-effective in a Norwegian setting.

**Conclusions:**

The results support the use of CIM as part of a public health program to improve physical activity and consequently avert morbidity and mortality. CIM is affordable and has a long-term effect on physical activity that in turn has a positive impact on health improvement.

**Supplementary Information:**

The online version contains supplementary material available at 10.1186/s12889-020-09764-5.

## Background

Studies suggest that cycle-network investments can increase cycling [1–3] and thereby improve health at the population level [4, 5]. The national and local governmental authorities in Norway have aimed to support the growth of cycling as a means of transportation with the aim to improve health through increased physical activity. Although building new cycling infrastructure is expensive compared to “soft measures” such as marketing campaigns for cycling promotion [6], improved cycling infrastructure makes cycling safer and more attractive.

The health sector services are increasingly focusing on providing more healthy years for all in Norway [7]. Thus, there is a need to prevent diseases and diminish the adverse impact of long-term medical conditions, and it was shown that physical activity plays a vital role in this endeavor [2, 8]. Physical inactivity increases the risk of lifestyle diseases and premature death. Specifically, physical inactivity is one of the leading modifiable risk factors of global mortality, with an estimated 20–30% increased annual risk of death compared to those who are physically active [9]. Further, physical inactivity was found to be independently responsible for several chronic diseases worldwide, including 6% of coronary heart disease (CHD), 7% of type 2 diabetes (T2D), 10% of breast cancer, and 10% of colon cancer cases [10]. Thus, promoting physical activity through cycling would have a wide range of potential health benefits in Norway, where the current cycling rate accounts for only 4% of total transportation journeys [11].

To our knowledge, there have been no studies on cost-effectiveness of cycle-networks construction in Norway. One cost-benefit analysis has been conducted on walking and cycling network investment initiatives in three Norwegian cities (Hokkusund, Hamar, and Trondheim), which utilizes statistical value-of-life methods to quantify the benefits of reduced mortality [12]. However, the health-benefit criterion for health economic evaluation is primarily concerned with health benefits measured in terms of quality-adjusted life-years (QALYs) gained, and can thus be applied to any type of intervention [7]. Cost-effectiveness studies of physical activity that simultaneously model the direct and indirect health benefits are sparse, and none is for Norway. In this study, we assess the cost-effectiveness of planned cycle-route investments in Oslo.

## Methods

### Change in cycling rates: regression analysis

We first address the effect of cycle network expansion on cycling rates. We estimated the change in the mode share of cycling resulting from cycling-network construction using a nonparametric regression method, which makes no assumptions about the functional form of the relationship between the outcome and the covariates. There is a lack of information on the association between the increase in cycle ridership and the construction of cycle roads constructed every year in Norway. Thus, data on cycling mode share and cycling network length were obtained for 123 major European cities with a population size of at least 100,000 from 11 European countries: Austria, Belgium, Denmark, France, Germany, Italy, the Netherlands, Spain, Sweden, Switzerland, and the United Kingdom [13] (Additional file 1). Data on cycling network lengths were retrieved from OpenStreetMap, and data on mode share and population size were obtained through the European Platform on Mobility Management Modal Split Tool [13–15]. The nonparametric regression was extracted using the modal share of cycling (in %) and cycle–networks length (in km per 100,000 population) as the independent and dependent variables, respectively. Using the fitted equation, we predicted the percentage of expansion in cycle ridership for every additional km of cycle length construction.

### Model structure: decision analytic model

We developed a Markov state-transition model that examines the cost-effectiveness of investing in cycle-network construction in Oslo. We refer to the model as the Cycle-network Investment Model (CIM), which was developed using the TreeAge Software (©TreeAge Pro 2020 (v2.1), Williamstown, MA, USA). Markov modeling is commonly used for the evaluation of long-term impact and cost-effectiveness of interventions. It is based on a number of mutually exclusive states into which the individual may or may not move at defined points in the future, such as being “physically active” or “physically inactive”. The model is re-evaluated at different points of time (cycles), and after each cycle, an individual may remain in the previous state or move to a different one depending on transition probabilities. Costs and outcomes (e.g. health) depend on the states and are calculated and cumulated after each cycle. Markov models are particularly well-suited to evaluate population-based health promotion efforts where costs and outcomes appear over an extended period of time [16].

**Fig. 1:**
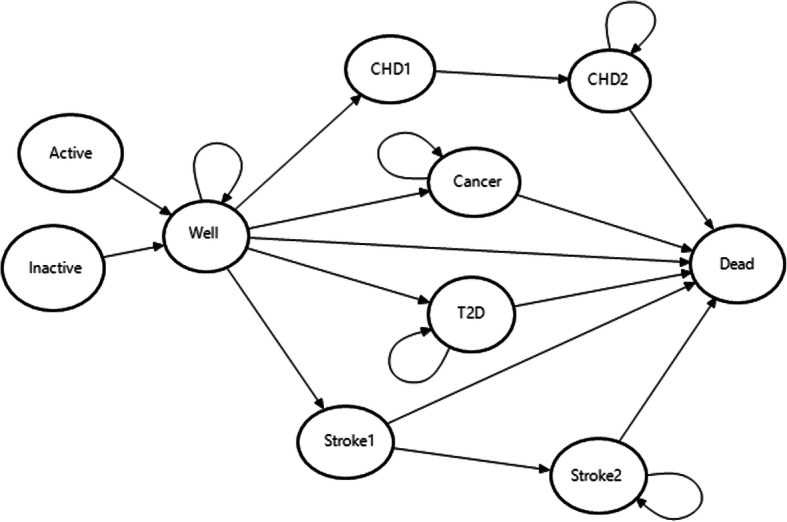
Markov transition model. *Note*: Circles represent Markov health states, and arrows indicate the transitions among these health states. *Death* is an absorbing state from which no future transitions are possible, and *Well* is a disease-free state from which the Markov process starts. *CHD1* fatal coronary heart disease, *CHD2* non-fatal coronary heart disease, *Stroke1* fatal stroke, *Stroke2* non-fatal stroke, *T2D* type 2 diabetes

Health outcomes and costs associated with the construction of new cycle networks were compared to the status quo (no intervention). Through CIM, we modeled the incidence of four disease events related to physical activity with and without additional cycling: CHD, stroke, T2D, and cancer. We assumed that only a portion of the cohort chooses cycling as their main means of transportation, and that all cyclists were considered physically active. We allowed for the possibility that some of the non-cyclists could be active elsewhere and achieve similar disease-risk profiles as the cyclists. The insufficiently active (hereafter inactive) group consisted of the portion of the population attaining less than 150 min of moderate-intensity physical activity per week [17]. The proportion of non-cyclists achieving the recommended physical activity level (28.6%) was obtained from the literature [18]. After cycle-network construction, individuals would settle into either “cyclists” or “non-cyclists”, the latter being further subdivided into the two states: “active” and “inactive”. The Markov model structure with possible transitions is illustrated in Fig. 1.

The risk of the four specified diseases declined over time for the “active” group. The starting age of the cohort was assumed at 30 years because of the low risk of non-communicable diseases due to physical inactivity before age 30 and the paucity of data on younger age groups. The time horizon of the CIM was assumed to be 25 years on the basis that active travel infrastructure might need rebuilding beyond such a horizon, and the model was evaluated using cycle lengths of one year. Future costs and health benefits were discounted at 4% per year [19]. Willingness-to-pay thresholds of 600,000 NOK ($72,550) were used to determine the likelihood of cost-effectiveness in all analyses, which is within the range commonly used in Norway [20, 21].

Initially, we assumed that everyone starts in the “Well” state (i.e. no disease). We assumed that health states included in the model were mutually exclusive, and members do not move between disease states. During each annual cycle, an individual has a risk of moving to one of the disease states or to the “Dead” state. Individuals with T2D and cancer are assumed to either stay in their states or die in the subsequent cycles. For CHD and stroke, we assumed that a given proportion of events would be immediately fatal and people who survived one of these events would have an increased subsequent risk of death. Individuals in the non-fatal states can stay in that state, or move to the “Dead” state. The “Dead” state is absorbing, meaning that no further transitions are possible.

### Disease risk and mortality

We conservatively assumed that the intervention of building new cycle-networks influences the incidence of disease only in healthy participants *at risk*. Initially, age-specific incidence rates of cancer, CHD, stroke, and T2D for the general Norwegian population were obtained [22, 23]. These incidence rates were then adjusted by population-attributable fractions [10, 24, 25] to obtain the probabilities of developing these conditions among the inactive individuals (Table 1). Finally, the probabilities of developing the specific diseases among active individuals were derived using relative-risk (RR) estimates identified from the literature (Table 2).

**Table 1:** Age-specific annual probabilities of experiencing the different health events

Age	Incidence [22, 23]				Mortality rate [26]		Case fatality rate [27, 28]	
	T2D	Cancer	Stroke	CHD	CVD	All-cause	CHD	Stroke
30–34	0.0016	0.0011	0.0003	0.0001	0.00002	0.00046	0.0877	0.2346
35–39	0.0018	0.0016	0.0005	0.0003	0.00005	0.00059	0.0877	0. 2346
40–44	0.0031	0.0022	0.0010	0.0006	0.0001	0.00082	0.0877	0. 2346
45–49	0.0050	0.0035	0.0017	0.0010	0.0002	0.00136	0.0877	0. 2346
50–54	0.0076	0.0058	0.0025	0.0018	0.0004	0.00231	0.0877	0. 2346
55–59	0.0098	0.0087	0.0033	0.0029	0.0007	0.00384	0.1155	0.2328
60–64	0.0106	0.0134	0.0044	0.0046	0.0011	0.00610	0.1155	0.2328
65–69	0.0105	0.0190	0.0056	0.0069	0.0020	0.01052	0.2107	0.2347
70+	0.0121	0.0252	0.0070	0.0102	0.0036	0.01742	0.2107	0.2347

**Table 2:** Relative risk of incidence and mortality for the disease states

Disease	Base value	Lower	Upper	Distribution	Source
	RR of mortality^a^				
CHD	3.89	3.81	3.97	Lognormal	[29]
Stroke	3.89	3.81	3.97	Lognormal	[29]
T2D	2.61	2.34	2.88	Lognormal	[30]
Cancer	4.20	4.00	4.30	lognormal	[31]
	Relative risk for disease (Active vs inactive)				
Cancer	0.55	0.36	0.84	Lognormal	[32]
CHD	0.80	0.75	0.86	Lognormal	[33]
Stroke	0.82	0.77	0.87	Lognormal	[33]
T2D	0.74	0.72	0.77	Lognormal	[33]

^**a**^Relative risks (RRs) of CVD mortality except for cancer (which is RR of all-cause mortality)

In the absence of direct probabilities for CVD-related and other mortality among individuals with CHD, stroke, and T2D, RRs for these conditions [27, 29, 30] were applied to adjust for age-specific mortality rates (Table 2). Age-specific mortality rates for CVD and all-cause mortality were retrieved from Norwegian life tables and cause of death registries [26, 34]. Mortality constituted disease-specific mortality and mortality due to other causes. Mortality due to other causes was given by all-cause mortality less mortality from the four disease conditions included in the model. Individuals with cancer were assigned an increased risk of mortality using data from the Finnish population based registry study [31]. Due to the lack of Norwegian-specific data, case fatality rates for CHD and stroke were taken from the international literature [27, 28]. Because we are mainly interested in the primary prevention of disease events, the mortality risk from these events (conditional on having the disease), except for T2D, was assumed to be independent of physical activity. For T2D, we also assumed that the risk of death declined over time at a faster rate for the active group than for the inactive group [35, 36].

### Cycle-network construction and cycle trips

The Oslo municipality has an ambitious plan to create a network of 530 km cycle infrastructure in two phases, where 70% of the costs are covered by the municipality and the rest by the state government. Phase I is a 100 km cycle-network to be built by 2025 (i.e., an increase from 180 km in 2015 to 280 km in 2025). The goal is to increase the share of cycling to 25% upon the completion of phase I, which is much higher than the base value of 6% [37].

### Costs

All costs in the CIM are expressed in 2017 US Dollars ($), and the annual average exchange rate is assumed to be 8.27 NOK per $1. All costs were defined as annual costs per person (Table 3). Cost estimations for the treatment of CHD and stroke were made according to methods described in the NorCaD model [19] and a stroke study in the Norwegian settings [39]. The treatment costs of T2D [40, 41] and cancer [42] were estimated from the literature in a Norwegian context.

**Table 3:** Cost and utility parameters

***Description of Costs and utilities***	***Cost ($) and utility values***	***SD***	***Distribution for sensitivity analysis***	***Source***
***Costs***				
Investment cost, total/per capita	3,022,975/4.5	1.690	Gamma	[38]
Maintenance cost, total/per capita	44,438/0.066	0.020	Gamma	[12]
Cost of CHD 1st event	22,133	8300	Gamma	[19]
Cost of post-CHD 1st event	21,597	8099	Gamma	[19]
Cost of stroke 1st event	25,421	9533	Gamma	[19]
Cost of post stroke 1st event	11,962	4486	Gamma	[39]
Cost of T2D	5247	1968	Gamma	[40, 41]
Cost of cancer	13,810	5179	Gamma	[42]
***Utilities***				
Healthy	1.00			Assumed
Cancer	0.74	0.015	Beta	[43]
CHD1	0.47	0.016	Beta	[28, 44]
CHD1+	0.56	0.016	Beta	[44]
Stroke1	0.50	0.036	Beta	[44]
Stroke1+	0.50	0.036	Beta	[44]
T2D	0.81	0.190	Beta	[45]
Wellbeing gain when active	0.05	0.013	Beta	[28]

The cost of intervention per capita was determined by dividing the total cycle-network investment costs in 2017 by the Oslo population size (666,759) in that year. This was assumed as a prior cost and thus was included in the intervention arm as a one-time cost. Following the Norwegian Public Roads Administration [38], an average construction cost of 25,000 NOK per meter of cycling networks was used. Following price adjustment and currency conversion, the construction cost was estimated at $3 million per km. In addition to construction costs, an annual maintenance cost of approximately 7% of the total investment cost was considered [37, 46].

### Outcome measures

QALYs are the primary outcome measure of the model and were obtained by weighting the time spent in the various health states by the utility values associated with each state. In the present study, health state utility values were measured by the EuroQol five-dimensional questionnaire (EQ-5D) and assigned to all disease states and the healthy state (https://euroqol.org/).

In addition to the direct health effect of cycling, increased physical activity is assumed to improve wellbeing. Only one study was identified that had estimated the relationship between the amount of physical activity and the score on the utility scale [47]. The mentioned study and an analysis performed by the National Institute for Health and Clinical Excellence on promoting physical activity [48] estimated that every 30 min of physical activity resulted in a QALY gain of 2.22 × 10^− 4^ due to improvements in wellbeing. We used this value to transform physical activity levels for the active group into QALYs, which amounted to a QALY gain of over 0.05 per year due to improvements in wellbeing (Table 3).

### Sensitivity analyses

To assess the robustness of the model results and how uncertainties around input values and assumptions might potentially influence them, we carried out scenario and sensitivity analyses. In the scenario analysis, we varied the time horizon of the model from the baseline of 25 years to 20 years and then to 30 years to assess the impact of the time horizon on the cost-effectiveness of the intervention. Scenario analyses for 35 and 40 years of time horizon have also been conducted to check the influence of higher relative risk of the described diseases at an older age. Further, given Oslo’s goal of a 25% mode share for cycling by the year 2025 [37], this study considered four scenarios of cycle mode shares: 11.5, 15, 20, and 25%. In addition, both one-way and probabilistic sensitivity analyses were conducted to explore the effects of key input parameters. In the probabilistic sensitivity analyses (with 10,000 iterations), cost data, probabilities/utilities, and relative-risk reductions were assumed to follow gamma, beta, and log-normal distributions, respectively. Gamma distributions are constrained on the interval 0 to infinity, and hence appropriate to represent uncertainty in cost parameters; beta distributions are constrained on the interval 0 to 1 and suitable to model uncertainties in probabilities or utilities; and RRs are ratios with confidence limits calculated on the log-scale, and hence log-normal is the appropriate distribution [16].

## Results

### Regression results

**Fig. 2:**
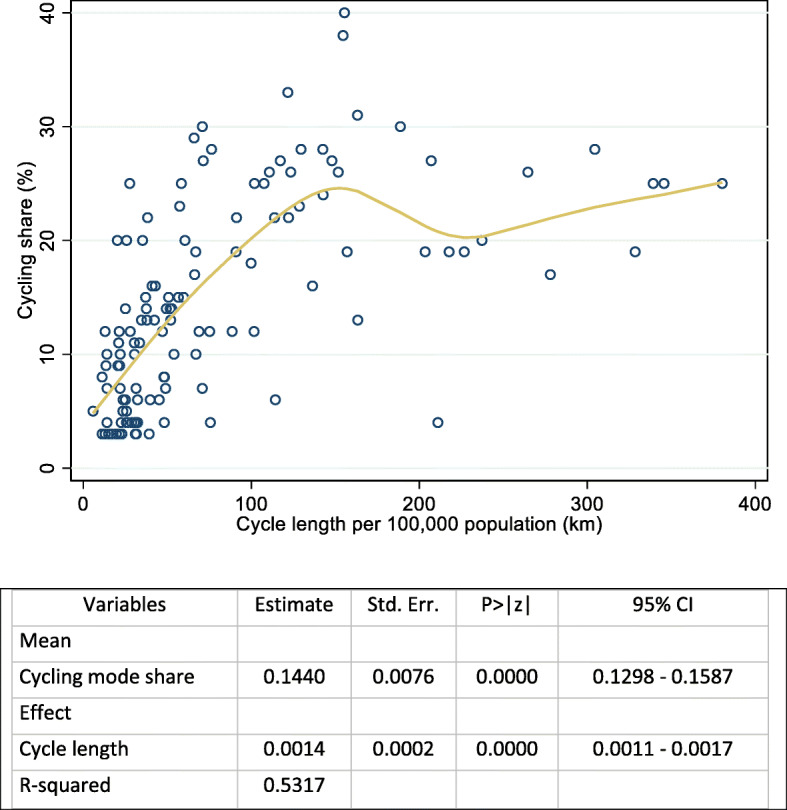
Nonparametric regression between cycle path length (in km per 100,000 population) and cycling mode share

The nonparametric regression results show that the average marginal effect of cycle path length is 0.14%; i.e., a one km increase in length of cycle paths leads to a 0.14% increase in cycle modal share (Fig. 2). Based on this model, the share of cycling would increase by 3% if Oslo’s cycle-network length increased from 180 km to 280 km as per Phase I of the cycle-network expansion plan in Oslo. After adjusting by the base-line value of cycling, the chance of cycling (modal share) after the cycle-network investment becomes 9%. Alternatively, using the fitted model, we predicted that the probability of cycling would increase by 11.5% if a total of 280 cycle-network kms were constructed in 2025. The conservative value of 9% cycling share is used in the base-case analysis. Other policies may increase the share further, but are not considered here.

### Cost-effectiveness analysis: base case analysis

The total costs, total QALYs, and incremental cost-effectiveness ratio for the CIM are presented in Table 4. Cycle-network construction as part of promoting physical activity produced an additional cost of $416 per person and yielded 0.019 QALYs per person over the life horizon of the intervention compared with the status quo. The incremental cost per QALY gained (or ICER) is $22,350 and thus can be considered highly cost-effective at a threshold of $72,550 per QALY.

**Table 4:** Cost-effectiveness results comparing CIM with status quo

CIM	Status quo		CIM		Incremental		ICER
	Cost	QALY	Cost	QALY	ΔCost($)	ΔQALY	($/QALY)
Base case	6875	15.240	7291	15.259	416	0.019	22,350
***Change cycle share***							
Predicted cycle share (11.5%)	6875	15.240	7220	15.273	345	0.033	10,292
Scenario 2 (15%)	6875	15.240	7188	15.297	313	0.057	5533
Scenario 3 (20%)	6875	15.240	7119	15.328	244	0.088	2766
Scenario 4 (25%)	6875	15.240	7060	15.359	185	0.119	1548
***Time horizon***							
Scenario 5 (20 years)	4155	13.198	4589	13.213	434	0.015	27,967
Scenario 6 (30 years)	10,366	16.864	10,760	16.885	394	0.021	18,536
Scenario 7 (35 years)	14,420	18.139	14,782	18.163	362	0.024	15,366
Scenario 8 (40 years)	18,788	19.121	19,110	19.147	323	0.026	12,368

*QALY* quality adjusted life year, *ICER* incremental cost-effectiveness ratio, *CIM* cycle-network investment model

### Scenario and sensitivity analyses

The scenario analyses (Table 4) revealed that the percentage share of cycling (following cycle-network investment) is a crucial parameter. If Oslo achieves its ambitious plan of 25% modal share for cycling through other forms of physical activity promotion, CIM alone would be cost-effective at $1548 per QALY (excluding other costs of achieving the planned share of 25% modal share). Further, reducing the life of the project from 25 to 20 years had a considerable effect: the incremental cost per QALY rose to $27,967. In contrast, increasing the life of the project by 10 years (on top of the base-case time horizon of 25 years) reduced the incremental cost per QALY gained to two-thirds of the base-case value. This substantial reduction in ICER is because the CIM’s incremental benefits (or QALY gains) continue to accumulate over an extended duration; at the same time, its incremental costs during the subsequent periods decline due to a reduction in the risk of disease events (Table 4).

**Fig. 3:**
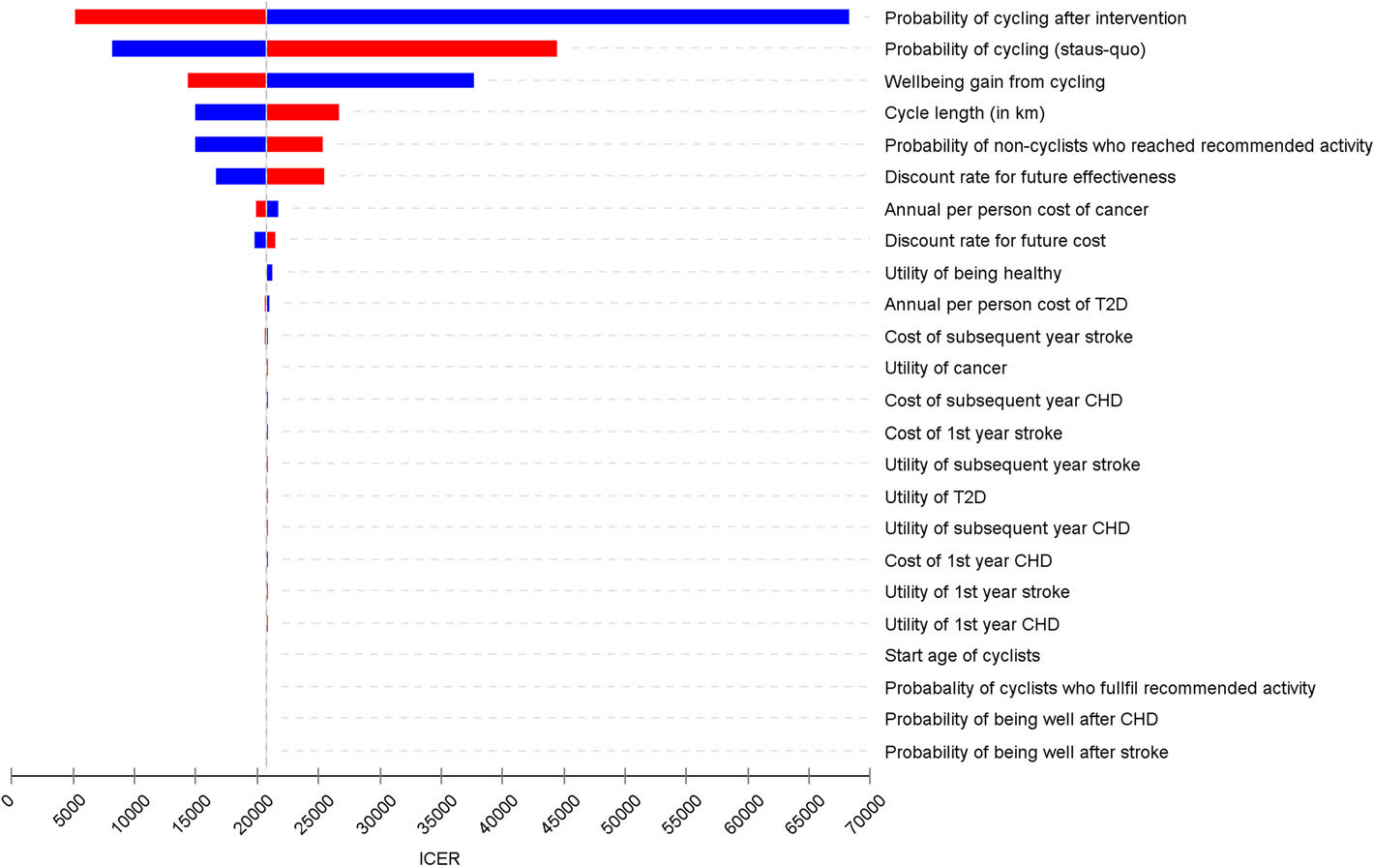
An influence analysis (Tornado diagram). *CHD* coronary heart disease, *T2D* type-2 diabetes, *ICER* incremental cost-effectiveness ratio. Each bar represents the range of outcomes produced when each input parameters is set to low (blue bar) and high values (red bar), with the other variables being held constant. The solid vertical line represents the value of the outcome when the baseline values are used for all input parameters. The upper bars represent input parameters that contribute most to the variability of the outcome

In the one-way sensitivity analysis, the most important variable is the share of cycling, followed by well-being from physical activity (Fig. 3). The lower the cycling share and the wellbeing gain, the higher the ICER. However, plausible changes to the assumed effectiveness of CIM led to ICER estimates that remained well below the threshold of $72,550 per QALY. The analysis is generally robust to variations in other key input parameters.

**Fig. 4:**
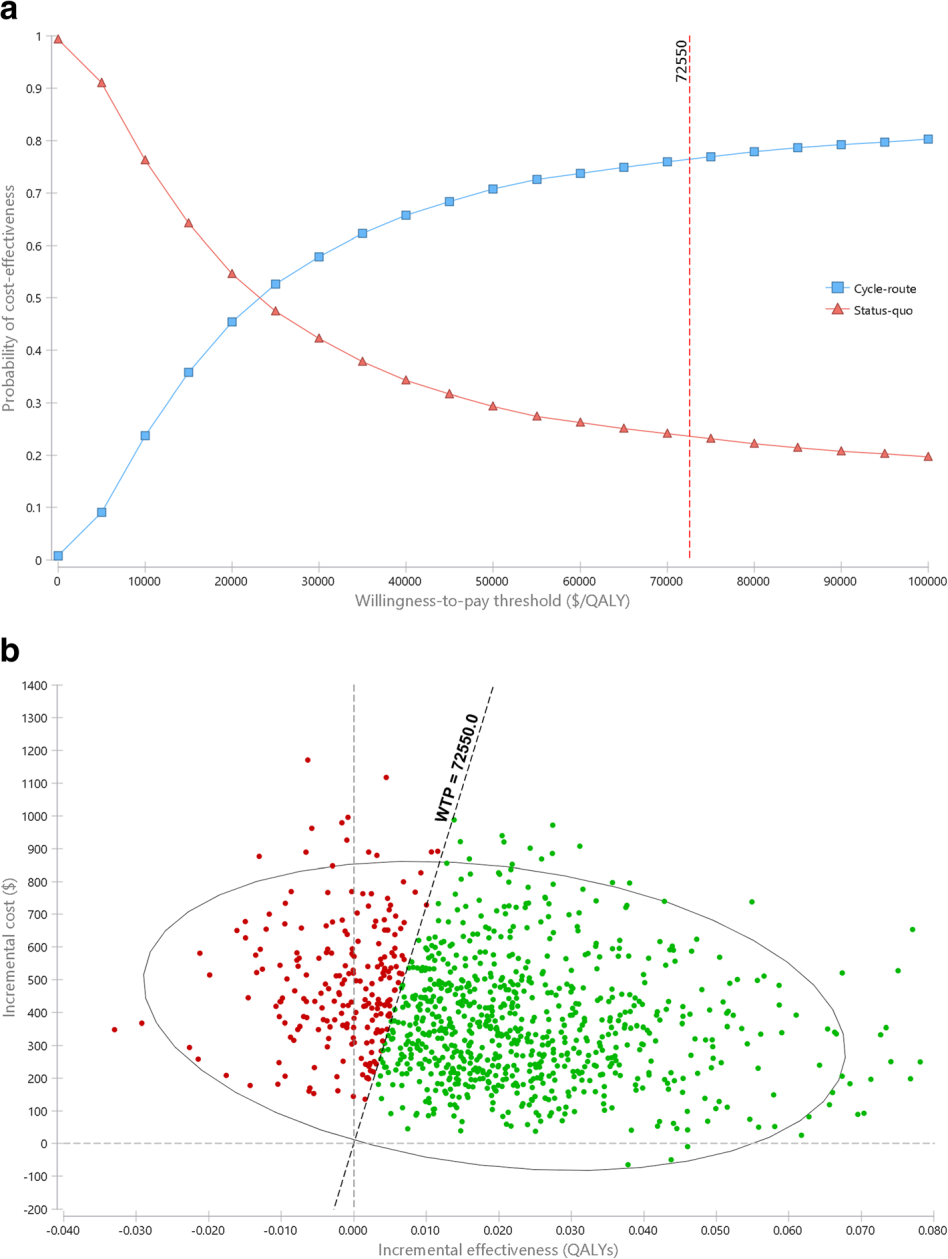
Cost-effectiveness acceptability curve and cost-effectiveness plane showing results for 10,000 Monte Carlo simulations

Figure 4 presents a cost-effectiveness acceptability curve and a cost-effectiveness plane for each of the 10,000 simulations. In general, the cost-effectiveness of cycle-network construction compared to the status quo depends on the decision-makers’ maximum willingness to pay per additional QALY. The probability that the CIM would be cost-effective at different thresholds of willingness to pay is depicted in Fig. 4a (results produced based on 10,000 Monte Carlo simulations). For example, the ICER for CIM compared with the status quo was considered cost effective at $30,000 per QALY, a threshold well below the commonly used value in Norwegian settings (shown by a $72,550 per QALY threshold line in Fig. 4b).

## Discussion

This study assessed the cost-effectiveness of new cycle-network investment using a Markov state-transition modeling approach. The cycle-network investment compared to the status quo is cost-effective as it is well below the suggested threshold of $72,550 per QALY, at which there is a 76% chance that the CIM will be cost-effective. Setting other benefits aside, health and wellbeing benefits of physical activity justify the investment in cycle infrastructure. This is more cost-effective than other health interventions provided in Norway such as acute ischemic stroke treatment with intravenous thrombolysis through ‘mobile stroke unit’, which costs nearly two times more (over $40,000 per QALY gained) [49].

Similar results have been found in previous studies on the cost-effectiveness of cycling infrastructure to improve physical activity, despite the difference in the methodological approaches used. Gu, et al. [50] found that investments in cycle lanes are more cost-effective than the majority of preventive approaches used nowadays. Other studies have also reported positive benefit-cost ratios [1, 12, 51]. Recently, there has been a growing need for health economic evaluations of such programs because they assist decision-makers in establishing priorities within cost-constrained health care budgets.

The base-case results were generally robust to probabilistic and deterministic sensitivity analyses. The ICERs modestly decline with an increase in the starting age of the cohort, indicating that the payoffs would be higher if the intervention is targeting older individuals. This makes sense because aging is the main risk factor for the development of multiple non-communicable diseases, including cardiovascular diseases, cancer and diabetes.

This study is timely because it is relevant to the ongoing public and political debate about public health interventions in Norway, and addresses the lack of economic evidence on public health interventions. Another strength is the quantitative analysis of wider health aspects (including wellbeing) associated with the cycling infrastructure. The use of a Markov model is also a strength because it represents processes that evolve over time, which is particularly suited for modelling the progression of chronic disease and interventions (e.g., cycling) that aim to reduce the risk or severity of diseases. However, the study has a number of limitations. Costs and utility data were taken from secondary sources. To minimize the effect of such limitations, data from Norway were used as much as possible. In the absence of such data, information from similar studies was carefully retrieved from similar countries and reported here with transparency. Further, uncertainty in the cost and utility estimates was incorporated and analyzed in the model through deterministic and probabilistic sensitivity analyses.

The model included only cycle-network investment and health care costs, and the results might vary if other costs and productivity changes are included. In addition, we might have underestimated the benefits of the program by considering that the intervention only modifies disease incidence, while it may also reduce the risk of mortality [52]. Uncertainty with regard to gain in QALY due to improvement in wellbeing resulting from increased share of cycling could be another limitation, and we might have overestimated the benefits if there is no or only trivial wellbeing gain. Furthermore, the assumption that all cyclists are active might not always hold because an increase in cycling might not translate directly into an equivalent increase in overall physical activity. Had only 90% of cyclists been assumed to be active, the incremental cost per QALY would have increased by 11%.

Other disease conditions that may be affected by physical activity were excluded from the analysis. Such assumptions might lead to underestimation of the cost-effectiveness of the CIM. The exclusion of adverse effects of cycling (e.g., injuries), and additional favorable effects of cycling (e.g., reduced air pollution and congestion as well as wear and tear of roadways) constitute another limitation. However, injuries are generally low and less likely to significantly affect the results at the population level [27]. Further, it has been argued that the net number of fatalities and crashes would not increase with investments in cycling [51]. The issue of air pollution is also dynamic in the sense that cyclists could potentially be exposed to higher doses of pollution even though cycling reduces overall pollution emissions. The base-case analysis assumed cost-effectiveness of cycle-networks investment for people starting to ride cycle at the age of 30 over the next 25 years (i.e., from age 30 to 55 years). This simplifying assumption likely yields conservative estimates, because prevalence of the diseases and preventable mortality increase among older age groups. We considered time horizons up to 40 years in scenario analyses, and found that cycle-network investment becomes more cost-effective with longer analytical horizons.

In cost-benefit analysis terms, the health benefits (only) measured in monetary terms (value of statistical life estimates), which are often used in the transport sector, would clearly be substantially larger than the investment costs. Thus, our study can reasonably justify the need for further investment in cycle-networks as a crucial part of improvements in health and wellbeing as well as having an efficient urban infrastructure.

## Conclusions

The findings of this study show that investments in cycle-network construction in Oslo would be cost-effective. Cycling can be done as part of daily travel routines, and thus has the potential to reduce the risk of a range of health conditions, mainly cancer, heart disease, T2D, and stroke – all of which are preventable causes of premature death. It can also delay mortality if such diseases develop. Our findings provide evidence that cycle-networks investment may help increase overall physical activity levels and thereby produce substantial health benefits. Thus, policy-makers should focus on placing cycling at the heart of a healthy transport policy making cycling a convenient, safe, and attractive everyday activity.

## Supplementary Information

**Additional file 1.** Cycling network and mode share data for 123 major European cities.

## Data Availability

The datasets generated and/or analyzed during the current study are available in OpenStreetMap and European Platform on Mobility Management (EPoMM) repository (openstreetmap.org and epomm.eu/tems/about_tems.phtml) and are included in this article as an online supplementary material.

## Abbreviations

CIM: Cycle-networks Investment Model
QALY: Quality-Adjusted Life Year
CHD: Coronary Heart Disease
T2D: Type 2 Diabetes
CVD: Cardiovascular Disease
EQ-5D: EuroQol 5-Dimensional Questionnaire
ICER: Incremental Cost-Effectiveness Ratio
RR: Relative Risk
