# Supplementary material for: Is cycle network expansion cost-effective? A health economic evaluation of cycling in Oslo
Source: BMC Public Health. 2020 Dec 7;20:1869. doi: 10.1186/s12889-020-09764-5 (PMC7720509; doi:10.1186/s12889-020-09764-5)
Supplement: Supplementary file 1 — Additional file 1. Cycling network and mode share data for 123 major European cities. [file 12889_2020_9764_MOESM1_ESM.docx]

**Additional file 1.** Cycling network and mode share data for 123 major European cities

| Country | City | Population^a^ | Cycling share (%)^a^ | Year | Cycle network (km)^b^ |
| --- | --- | --- | --- | --- | --- |
| Austria | Salzburg | 145 700 | 20 | 2012 | 51.38 |
| Austria | Linz | 190 000 | 5 | 2008 | 48.81 |
| Austria | Wien | 1 797 340 | 6 | 2015 | 715.63 |
| Austria | Graz | 257 328 | 14 | 2013 | 127.09 |
| Belgium | Brussels | 1 139 000 | 3 | 2010 | 261.25 |
| Belgium | Antwerp | 493 517 | 23 | 2011 | 283.1 |
| Belgium | Ghent | 251 000 | 20 | 2012 | 64.7 |
| Belgium | Brugge | 117 000 | 28 | 2011 | 89.31 |
| Denmark | Odense | 187 000 | 27 | 2008 | 387.24 |
| Denmark | Copenhagen | 591 000 | 30 | 2014 | 418.74 |
| France | Grenoble | 393 000 | 4 | 2010 | 55.35 |
| France | Bordeaux | 881 000 | 3 | 2009 | 132.14 |
| France | Strasbourg | 439 000 | 8 | 2009 | 211.95 |
| France | Nantes | 580 000 | 5 | 2012 | 136.53 |
| France | Tours | 348 000 | 4 | 2008 | 78.45 |
| France | Rennes | 392 000 | 4 | 2007 | 124.44 |
| France | Paris | 2 211 300 | 3 | 2008 | 287.07 |
| Germany | Heidelberg | 139 200 | 25 | 2010 | 38.46 |
| Germany | Bremen | 547 735 | 25 | 2008 | 589.29 |
| Germany | Bochum | 374 737 | 6 | 2010 | 88.62 |
| Germany | Mannheim | 326 964 | 13 | 2008 | 138.35 |
| Germany | Bielefeld | 323 270 | 15 | 2010 | 182.96 |
| Germany | Reutlingen | 112 258 | 15 | 2007 | 56.97 |
| Germany | München | 1 326 810 | 14 | 2008 | 698.68 |
| Germany | Kaiserslautern | 105 352 | 3 | 2008 | 41.28 |
| Germany | Witten | 100 248 | 3 | 2006 | 21.56 |
| Germany | Nürnberg | 506 000 | 11 | 2011 | 106.37 |
| Germany | Potsdam | 149 687 | 20 | 2008 | 90.56 |
| Germany | Heilbronn | 122 302 | 8 | 2008 | 13.69 |
| Germany | Halle | 233 000 | 12 | 2010 | 30.18 |
| Germany | Magdeburg | 234 977 | 10 | 2008 | 157.52 |
| Germany | Ulm | 121 648 | 9 | 2008 | 16.39 |
| Germany | Frankfurt | 680 000 | 11 | 2008 | 227.57 |
| Germany | Chemnitz | 249 500 | 6 | 2008 | 80.55 |
| Germany | Saarbrücken | 175 741 | 4 | 2010 | 45.07 |
| Germany | Hamm | 182 459 | 17 | 2008 | 120.9 |
| Germany | Offenbach am Main | 120 500 | 9 | 2008 | 26.04 |
| Germany | Darmstadt | 143 499 | 15 | 2011 | 53.2 |
| Germany | Köln | 989 766 | 12 | 2006 | 211.56 |
| Germany | Hamburg | 1 735 660 | 12 | 2008 | 817 |
| Germany | Mainz | 201 500 | 10 | 2008 | 61.32 |
| Germany | Osnabrück | 164 405 | 12 | 2010 | 167.14 |
| Germany | Aachen | 249 000 | 12 | 2012 | 171.56 |
| Germany | Leipzig | 515 418 | 14 | 2008 | 266.44 |
| Germany | Berlin | 3 506 240 | 13 | 2008 | 1327.3 |
| Germany | Erfurt | 201 368 | 8 | 2008 | 96.59 |
| Germany | Münster | 280 000 | 38 | 2007 | 432.91 |
| Germany | Halle (Saale) | 238 837 | 14 | 2008 | 89.59 |
| Germany | Jena | 110 097 | 10 | 2008 | 24.18 |
| Germany | Hildesheim | 103 593 | 12 | 2007 | 91.78 |
| Germany | Solingen | 161 779 | 3 | 2008 | 51.05 |
| Germany | Stuttgart | 592 915 | 5 | 2009 | 34.26 |
| Germany | Hannover | 518 386 | 19 | 2011 | 471.08 |
| Germany | Dresden | 512 546 | 16 | 2008 | 209.71 |
| Germany | Karlsruhe | 298 000 | 25 | 2012 | 173.93 |
| Germany | Augsburg | 265 000 | 13 | 2010 | 91.7 |
| Germany | Oldenburg | 159 563 | 22 | 2007 | 145.5 |
| Germany | Kassel | 198 167 | 7 | 2008 | 43.44 |
| Germany | Gera | 105 689 | 4 | 2008 | 27.8 |
| Germany | Göttingen | 123 594 | 27 | 2009 | 144.93 |
| Germany | Düsseldorf | 629 005 | 11 | 2008 | 190.95 |
| Germany | Neuss | 153 758 | 10 | 2012 | 83 |
| Germany | Bonn | 317 949 | 12 | 2008 | 88.75 |
| Italy | Parma | 187 214 | 6 | 2010 | 84.58 |
| Italy | Padua | 205 631 | 16 | 2011 | 88.07 |
| Italy | Verona | 265 368 | 9 | 2008 | 54.65 |
| Italy | Ferrara | 135 000 | 27 | 2008 | 96.36 |
| Italy | Reggio Emilia | 163 928 | 18 | 2012 | 163.71 |
| Italy | Bologna | 373 026 | 7 | 2007 | 117.07 |
| Italy | Bolzano | 104 000 | 29 | 2009 | 68.58 |
| Italy | Ravenna | 154 288 | 15 | 2012 | 92.07 |
| Netherlands | Eindhoven | 220 782 | 40 | 2014 | 343.23 |
| Netherlands | Arnhem | 143 582 | 19 | 2008 | 312.64 |
| Netherlands | Ede | 107 686 | 25 | 2008 | 409.55 |
| Netherlands | Groningen | 182 484 | 31 | 2008 | 297.82 |
| Netherlands | Emmen | 109 151 | 25 | 2008 | 377.31 |
| Netherlands | Apeldoorn | 155 108 | 28 | 2008 | 472.27 |
| Netherlands | Nijmegen | 161 251 | 24 | 2008 | 230.2 |
| Netherlands | Haarlemmermeer | 140 648 | 17 | 2008 | 391.01 |
| Netherlands | Maastricht | 118 004 | 22 | 2008 | 144.16 |
| Netherlands | Amersfoort | 141 211 | 28 | 2008 | 201.31 |
| Netherlands | Zaanstad | 142 863 | 25 | 2008 | 145.54 |
| Netherlands | Enschede | 154 753 | 26 | 2008 | 234.84 |
| Netherlands | Breda | 180 053 | 27 | 2013 | 266.45 |
| Netherlands | Almere | 183 270 | 19 | 2008 | 415.59 |
| Netherlands | Utrecht | 316 000 | 26 | 2012 | 390.18 |
| Netherlands | Haarlem | 147 640 | 26 | 2008 | 163.52 |
| Netherlands | 's-Hertogenbosch | 136 481 | 19 | 2008 | 277.72 |
| Netherlands | Leiden | 116 878 | 33 | 2008 | 142.3 |
| Netherlands | Zoetermeer | 119 504 | 19 | 2008 | 187.67 |
| Netherlands | Amsterdam | 747 093 | 22 | 2008 | 850.29 |
| Netherlands | Tilburg | 202 091 | 23 | 2008 | 259.9 |
| Netherlands | Rotterdam | 582 951 | 16 | 2008 | 795.31 |
| Netherlands | Zwolle | 116 365 | 30 | 2008 | 219.61 |
| Spain | Valencia | 786 189 | 4 | 2012 | 205 |
| Spain | Burgos | 178 500 | 4 | 2010 | 57.65 |
| Spain | San Sebastián | 186 185 | 4 | 2011 | 56.85 |
| Spain | Sevilla | 702 355 | 6 | 2011 | 175.8 |
| Spain | Vitoria-Gasteiz | 240 000 | 13 | 2014 | 124.76 |
| Sweden | Norrköping | 129 985 | 13 | 2010 | 212.37 |
| Sweden | Lund | 110 332 | 26 | 2007 | 291.99 |
| Sweden | Uppsala | 140 175 | 28 | 2010 | 181.64 |
| Sweden | Malmö | 313 000 | 22 | 2013 | 119.68 |
| Sweden | Linköping | 141 200 | 20 | 2008 | 335.1 |
| Sweden | Gothenburg | 543 000 | 7 | 2014 | 265.89 |
| Sweden | Umeå | 110 000 | 19 | 2006 | 361.35 |
| Sweden | Örebro | 138 952 | 25 | 2012 | 471.35 |
| Switzerland | Basel | 169 464 | 20 | 2010 | 34.3 |
| Switzerland | Zürich | 410 404 | 4 | 2015 | 118.36 |
| UK | Southampton | 234 100 | 3 | 2010 | 46 |
| UK | Nottingham | 303 900 | 4 | 2011 | 146.51 |
| UK | York | 197 800 | 12 | 2011 | 148.76 |
| UK | London | 8 673 710 | 3 | 2015 | 969.17 |
| UK | Bristol | 437 000 | 14 | 2013 | 109.5 |
| UK | Peterborough | 164 000 | 6 | 2008 | 187.47 |
| UK | Swindon | 184 000 | 7 | 2010 | 130.34 |
| UK | Plymouth | 256 700 | 3 | 2010 | 79.42 |
| UK | Milton Keynes | 195 687 | 4 | 2009 | 413.05 |
| UK | Norwich | 132 512 | 10 | 2011 | 18.6 |
| UK | Portsmouth | 442 252 | 7 | 2008 | 62.38 |
| UK | Oxford | 150 200 | 19 | 2011 | 100.52 |
| UK | Southend-on-Sea | 174 300 | 3 | 2011 | 29.15 |
| UK | Exeter | 118 000 | 4 | 2008 | 89.25 |

^a^Population and cycling share data were retrieved from European Platform on Mobility Management (EPoMM, 2011). ^b^Cycling network length were obtained from OpenStreetMap (OSM) (OpenStreetMap contributors, 2019).
